# Quantification of neurofilament light and glial fibrillary acidic protein in finger-prick blood

**DOI:** 10.1093/braincomms/fcae151

**Published:** 2024-04-29

**Authors:** Magdalena A Kolanko, Hanna Huber, Michael C B David, Laia Montoliu-Gaya, Joel Simrén, Kaj Blennow, Henrik Zetterberg, Ramin Nilforooshan, Paresh Malhotra, David J Sharp, Nicholas J Ashton, Neil S N Graham

**Affiliations:** UK Dementia Research Institute Centre for Care Research and Technology, Imperial College London, 9SMUB, White City Campus, W12 0BZ London, UK; Department of Brain Sciences, Imperial College London, W12 0BZ London, UK; Department of Psychiatry and Neurochemistry, Institute of Neuroscience and Physiology, the Sahlgrenska Academy at the University of Gothenburg, 43141 Mölndal, Sweden; UK Dementia Research Institute Centre for Care Research and Technology, Imperial College London, 9SMUB, White City Campus, W12 0BZ London, UK; Department of Brain Sciences, Imperial College London, W12 0BZ London, UK; Department of Psychiatry and Neurochemistry, Institute of Neuroscience and Physiology, the Sahlgrenska Academy at the University of Gothenburg, 43141 Mölndal, Sweden; Department of Psychiatry and Neurochemistry, Institute of Neuroscience and Physiology, the Sahlgrenska Academy at the University of Gothenburg, 43141 Mölndal, Sweden; Clinical Neurochemistry Laboratory, Sahlgrenska University Hospital, Mölndal 43180, Sweden; Department of Psychiatry and Neurochemistry, Institute of Neuroscience and Physiology, the Sahlgrenska Academy at the University of Gothenburg, 43141 Mölndal, Sweden; Clinical Neurochemistry Laboratory, Sahlgrenska University Hospital, Mölndal 43180, Sweden; Department of Psychiatry and Neurochemistry, Institute of Neuroscience and Physiology, the Sahlgrenska Academy at the University of Gothenburg, 43141 Mölndal, Sweden; Clinical Neurochemistry Laboratory, Sahlgrenska University Hospital, Mölndal 43180, Sweden; Department of Neurodegenerative Disease, UCL Institute of Neurology, Queen Square, WC1N 3BG London, UK; UK Dementia Research Institute at UCL, WC1N 3BG London,UK; Hong Kong Center for Neurodegenerative Diseases, Clear Water Bay, Hong Kong, China; Wisconsin Alzheimer’s Disease Research Center, University of Wisconsin School of Medicine and Public Health, University of Wisconsin-Madison, Madison, 53792 WI, USA; UK Dementia Research Institute Centre for Care Research and Technology, Imperial College London, 9SMUB, White City Campus, W12 0BZ London, UK; Surrey and Borders Partnership NHS Foundation Trust, Leatherhead, KT22 7AD Surrey, UK; University of Surrey, GU2 7XH Guildford, UK; UK Dementia Research Institute Centre for Care Research and Technology, Imperial College London, 9SMUB, White City Campus, W12 0BZ London, UK; Department of Brain Sciences, Imperial College London, W12 0BZ London, UK; UK Dementia Research Institute Centre for Care Research and Technology, Imperial College London, 9SMUB, White City Campus, W12 0BZ London, UK; Department of Brain Sciences, Imperial College London, W12 0BZ London, UK; Centre for Injury Studies, Imperial College London, W12 0BZ London, UK; Department of Psychiatry and Neurochemistry, Institute of Neuroscience and Physiology, the Sahlgrenska Academy at the University of Gothenburg, 43141 Mölndal, Sweden; Institute of Psychiatry, Psychology and Neuroscience Maurice Wohl Institute Clinical Neuroscience Institute, King's College London, SE5 9RT London,UK; NIHR Biomedical Research Centre for Mental Health and Biomedical Research Unit for Dementia at South London and Maudsley NHS Foundation, SE5 8AF London, UK; Centre for Age-Related Medicine, Stavanger University Hospital, 4011 Stavanger, Norway; UK Dementia Research Institute Centre for Care Research and Technology, Imperial College London, 9SMUB, White City Campus, W12 0BZ London, UK; Department of Brain Sciences, Imperial College London, W12 0BZ London, UK; Centre for Injury Studies, Imperial College London, W12 0BZ London, UK

**Keywords:** dementia, biomarkers, head injury, testing, diagnosis

## Abstract

An accurate diagnosis of neurodegenerative disease and traumatic brain injury is important for prognostication and treatment. Neurofilament light and glial fibrillary acidic protein (GFAP) are leading biomarkers for neurodegeneration and glial activation that are detectable in blood. Yet, current recommendations require rapid centrifugation and ultra-low temperature storage post-venepuncture. Here, we investigated if these markers can be accurately measured in finger-prick blood using dried plasma spot cards. Fifty patients (46 with dementia; 4 with traumatic brain injury) and 19 healthy volunteers underwent finger-prick and venous sampling using dried plasma spot cards and aligned plasma sampling. Neurofilament light and GFAP were quantified using a Single molecule array assay and correlations between plasma and dried plasma spot cards assessed. Biomarker concentrations in plasma and finger-prick dried plasma spot samples were significantly positively correlated (neurofilament light ***ρ*** = 0.57; GFAP ***ρ*** = 0.58, *P* < 0.001). Finger-prick neurofilament light and GFAP were significantly elevated after acute traumatic brain injury with non-significant group-level increases in dementia (91% having Alzheimer’s disease dementia). In conclusion, we present preliminary evidence that quantifying GFAP and neurofilament light using finger-prick blood collection is viable, with samples stored at room temperature using dried plasma spot cards. This has potential to expand and promote equitable testing access, including in settings where trained personnel are unavailable to perform venepuncture.

## Teaser

Kolanko *et al*. show that concentrations of fluid biomarkers neurofilament light and GFAP in finger-prick blood (using dried plasma spot cards) correlate well with levels in plasma, the gold standard. This finding has important implications in widening access to testing for neurological conditions including neurotrauma and dementia.

## Graphical Abstract

**Graphical Abstract:**
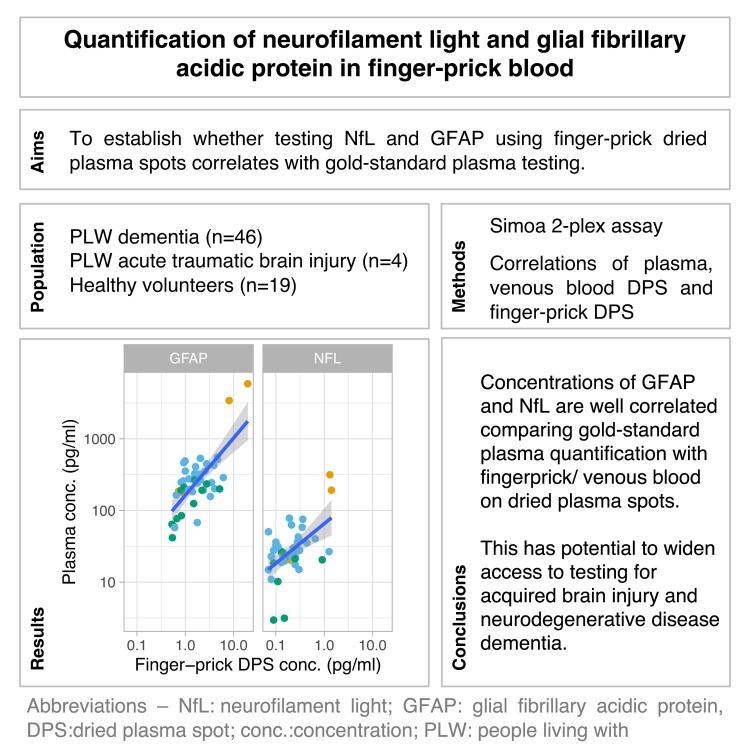

## Introduction

Recent technological advances in the analysis of fluid biomarkers allow quantification of axonal cytoskeletal protein neurofilament light (NfL) and astroglial marker glial fibrillary acidic protein (GFAP) in venous blood, reducing the need for cerebrospinal fluid sampling for diagnostic confirmatory analyses. These blood markers are now considered reliable measures of neuronal and astrocytic damage respectively, as a consequence of neurodegenerative,^1,2^ traumatic,^3,4^ cardiac arrest^5^ and neuroinflammatory states,^6^ with putative roles in screening, diagnosis, activity monitoring and assessment of treatment-response. In the context of Alzheimer’s disease (AD), GFAP has been shown to increase concurrently with amyloid-PET signal in the brain^7^ and increase >10 years prior to estimated disease onset in genetically determined AD.^8,9^ While these advances are opening new diagnostic avenues in clinical disciplines ranging from internal medicine to psychiatry and neurosurgery, there remain logistical challenges which could potentially reduce clinical impact. Currently, samples must be taken using venepuncture which typically requires a healthcare professional to be performed. Once taken, samples typically need to be centrifuged within a few hours and plasma extracted and either shipped to the laboratory for analyses in dry ice, or stored, typically at ultra-low temperatures.^10^

Dry plasma spot (DPS) cards can separate soluble proteins from the cell fraction of whole blood, equivalent to the separation of standard bottled blood achieved using centrifuges, without the issues of protein concentration variation with haematocrit in dried blood spot cards. A small volume of blood, such as from a finger-prick sample may be applied to a DPS card, negating the need for formal venepuncture. Cards show good stability when stored for up to 6 months at room temperature prior to analysis (Personal Communication, Dr Nick Ashton, Univ. Gothenburg). Other work by this group, currently under review, showed NfL levels from DPS remained stable over the 6 months period and although for GFAP a decrease was seen with time, the correlation with plasma levels was still strong and significant. The ability to sample neurodegeneration-associated proteins in a quick, minimally invasive way, including in non-clinical settings (such as patients’ homes) has significant implications for equity to diagnostics and great potential to improve personalized patient management.

Previous investigations in small numbers of patients with unselected clinical blood samples (i.e. without known neurodegenerative diagnosis, *n* = 30) or amyotrophic lateral sclerosis (*n* = 22 patients) suggest good correlations between venous blood DPS and plasma NfL (R^2^ = 0.95^11,12^ and R^2^ = 0.9^11,12^). However, finger-prick DPS blood quantification of neurodegeneration markers has not been published in the setting of AD dementia or traumatic brain injury (TBI), or in any population using GFAP, which has distinct and clinically useful biological characteristics, such as a rapid response to neurological insults and shorter half-life.^4^ In this study, we aimed to compare gold-standard plasma quantification of NfL and GFAP with finger-prick DPS testing in patients with dementia, acute TBI and healthy volunteers. We hypothesized, first, that plasma and finger-prick samples (as well as plasma and venous DPS samples) would show a strong positive correlation in respect of GFAP and NfL; and second, that finger-prick DPS biomarker concentrations would be significantly higher in acute TBI, and people with dementia compared with healthy volunteers.

## Materials and methods

Patient samples were analysed across several research studies. People with dementia (*n* = 27) were recruited from an ongoing longitudinal home-monitoring cohort study ‘MINDER’ run by the UK Dementia Research Institute Care Research and Technology Centre at Imperial College London. A further group of patients with dementia (*n* = 19) and healthy volunteers were recruited from an ongoing trial of noradrenergic add-on therapy with extended-release Guanfacine in Alzheimer's disease dementia (NorAD) (clinicaltrials.gov/ct2/show/NCT03116126). For inclusion, patients required diagnosis of AD per the National Institute of Neurological and Communicative Disorder and Stroke and Alzheimer’s Disease and Related Disorders Association (NINCDS-ADRDA) criteria, which do not necessitate biomarker confirmation.^13^ Patients were no longer taking the investigational medicinal products at the time of blood sample collection. Participants with dementia underwent Addenbrooke’s Cognitive Examination^14^ (version III) on the day of the blood test, except in a subset where this was linearly imputed from Alzheimer's Disease Assessment Scale–Cognitive Subscale-14, within 6 months of the blood test according to standard approaches.^15,16^ Patients within 10 days of acute moderate-severe TBI were recruited from the major trauma centre at Imperial College Healthcare NHS Trust, with severity classified by the Mayo severity classification.^17^ All participants provided written informed consent. Research ethics approvals were granted by the relevant local committees.

Venous blood was sampled peripherally and collected using ethylenediaminetetraacetic acid-coated tubes for plasma. After 30 min at room temperature, samples were centrifuged at 2500 *g* at 4°C for 20 min, then transferred into 1.4 mL aliquots, and frozen at −80°C. Matched DPS were collected using Telimmune™ Plasma Prep Duo Cards, a micro-sampling tool for plasma collection (Shimadzu, JP).^18^ The DPS were produced from venous blood sampled by venepuncture and from a paired finger-prick blood sample, obtained using a single-use safety lancet by a clinician (Unistik 3, 30G). Two drops of venous and finger-prick blood (approximately 60 µL) were spotted onto each Telimmune Duo card, producing two DPS per card. The cards were left to dry at room temperature for 15 min. They were then placed back in their original foil pouches containing a silica desiccant and stored at room temperature until analysis (up to 15 months). Cards were stored at room temperature with samples analysed a mean of 9.7 months (SD 2.5) after collection.

NfL and GFAP concentrations were measured using a 2-plex assay on a Quanterix Single molecule array (Simoa) HD-X analyser in three aligned samples for each individual: Tellimune finger-prick DPS, Tellimune venous DPS, and matched plasma samples. Calibrators were run in duplicates and obvious outlier calibrator replicates were masked before curve fitting. Plasma samples were diluted 4-fold and run in singlicates. Tellimune cards were eluted in an in-house developed propriety salt-based buffer (using 160 µL per dry blood spot sample) and run in singlicates. Saturated Tellimune filters were incubated while shaking at 37°C in a membrane embedded 96-well plate. The sample elution was performed by centrifugation at 2500 *g* directly into Simoa loading plate. Two quality control (QC) levels were run in duplicates in the beginning and the end of each run. In relation to NfL, for a QC sample with a concentration of 16.1 pg/mL, repeatability was 4.2% and intermediate precision was 4.2%; for a QC sample with a concentration of 91.2 pg/mL, repeatability was 5.2% and intermediate precision was 5.2%. In relation to GFAP, for a QC sample with a concentration of 117 pg/mL, repeatability was 5.8% and intermediate precision was 10.9%; for a QC sample with a concentration of 337 pg/mL, repeatability was 9.7% and intermediate precision was 14.7%.

Quantification within a Tellimune card failed in one healthy volunteer and one patient with AD, due to technical error. Results were not reported for Tellimune cards in 4 healthy volunteers where the GFAP concentration was undetectable and in one card where the NfL concentration was undetectable. Quantifiable results below the lower limit of quantification (0.8 pg/mL for NFL and 16.6 pg/mL for GFAP) were included in analyses.

## Statistical analysis

R version 4.2.2 (2022-10-31)/Rstudio 2022.12.0 + 353 were used for statistical analysis. Data were inspected for normality and non-parametric tests used as needed for comparing group differences of non-normal distributions. Chi-squared test was used to assess sex differences by group, and the Kruskal test for age, using Dunn’s post hoc test (Benjamini–Hochberg method for *P*-value adjustment for multiple comparisons). Spearman’s correlations were calculated between DPS samples and plasma. Linear mixed effects models (R package nlme) accounting for individual (random effect) were used to test how biomarker concentrations varied depending on biofluid (i.e. across plasma, finger-prick DPS and venous DPS). Linear regression was used to test the variation in plasma concentration by participant group, with concentration log-transformed and with age as a confounder. Using a two-tailed Fisher’s exact test with a bivariate normal model, a minimum of 29 patients is required to detect a significant correlation (expected coefficient = 0.5, beta = 0.2, two-tailed alpha = 0.05) of DPS and plasma; hence the study was well powered to detect this outcome.^19^ A sensitivity analysis was performed to assess the potential relationship between length of storage time before analysis, and biomarker concentrations, using linear regression (with biomarker concentration logged and age as a confounder).

## Results

A total of 46 patients with dementia, 4 with acute TBI and 19 healthy volunteers underwent neurodegeneration biomarker assessment (Table 1). Of the patients, AD dementia was the most common diagnosis in *n* = 42 (91% of the dementia group). There was no significant difference in sex by group (*P* > 0.05). There was no significant difference in age between TBI and healthy controls, but patients with dementia were significantly older than healthy controls (Z = 2.9, *P* = 0.005). Hence, age was included as a covariate in subsequent regressions assessing biomarkers by group. Patients with dementia were typically 5.8 years following diagnosis (mean, SD 3.6). The average score on Addenbrooke’s Cognitive Assessment was 64.9 (SD 18.6), where the maximum possible score is 100 and a mild/moderate-severe cut-off of 61 points is used.^20^ Four patients with acute moderate-severe TBI were assessed within 10 days of injury, all of whom had focal acute trauma-related pathologies on CT. Demographics including age and sex, by group, are described in detail in Table 1.

**Table 1:** Demographics and clinical characteristics

	Healthy volunteers	Acute TBI	Dementia
*N*	19	4	46
Age, mean (SD)	56.6, (22.4)	30.2, (6.4)	76.4, (7.5)
Sex, male, *N* (%)	10 (52.6%)	3 (75.0%)	26 (56.5%)
Clinical characteristics	—	Moderate-severe TBI (*n* = 4); time since injury mean 4.3 days (SD 4.9); focal traumatic CT abnormalities (*n* = 4); aetiology: fall (*n* = 2), RTA (*n* = 2)	ACE total score mean 64.9 (SD 18.6); years since diagnosis mean 5.8 (SD 3.6); aetiology AD (*N* = 42), FTD (*N* = 1), DLB (*N* = 1), VaD (*N* = 1), Mixed VaD/AD (*N* = 1)

Demographics and clinical characteristics of study population. TBI, traumatic brain injury; TBI severity indicated by Mayo classification; focal traumatic abnormalities defined CT findings including any of: extradural haematoma, subdural haematoma, subarachnoid blood, contusion. Dementia severity indexed using Addenbrooke’s Cognitive Assessment (ACE). AD, Alzheimer’s dementia; FTD, frontotemporal dementia; DLB, dementia with Lewy Bodies; VaD, vascular dementia; RTA, road traffic accident; SD, standard deviation.

Concentrations of fluid biomarkers were quantified across plasma, venous DPS and finger-prick DPS (Table 2). First, using linear mixed effects modelling (with subject as a random effect) we clarified the differences in each biomarker’s concentration by sample type. GFAP concentrations were substantially lower on DPS, for both finger-prick (reduced by 99.4% [95% CI 99.3–99.5]) and venous blood (by 99.0% [98.9–99.2]), compared with plasma. Similarly, for NfL, DPS concentrations were significantly lower on both finger-prick (by 99.3% [99.2–99.5]) and venous samples (99.1% [98.8–99.2]) versus plasma.

**Table 2:** Fluid biomarker concentrations in healthy controls and patients

	Healthy controls	Acute TBI	Dementia
GFAP concentration, pg/ml, median (IQR, *N*)			
Plasma	125 (61.9–208.0, *n* = 19)	1802.5 (183.8–4031.2, *n* = 4)	311.5 (209.2–426.0, *n* = 46)
Venous	1.7 (1.2–2.2, *n* = 14)	9.7 (1.3–24.3, *n* = 4)	2.6 (1.4–3.6, *n* = 44)
Finger-prick	0.8 (0.5–1.5, *n* = 17)	4.5 (0.9–11.0, *n* = 4)	1.7 (1.1–2.6, *n* = 32)
NfL concentration, pg/ml, median (IQR, *N*)			
Plasma	10.2 (4.9–20.6), *n* = 19)	106.2 (18.4–222.8 *n* = 4)	29 (22.6–39.0, *n* = 46)
Venous	0.2 (0.1–0.2, *n* = 17)	1.2 (0.1–2.5), *n* = 4)	0.2 (0.1–0.3, *n* = 44)
Finger-prick	0.1 (0.0–0.1, *n* = 16)	0.8 (0.2–1.3, *n* = 4)	0.2 (0.1–0.3, *n* = 32)

Biomarker concentrations in different bio-fluids. TBI, traumatic brain injury; NfL, neurofilament light; GFAP, glial fibrillary acidic protein; IQR, interquartile range.

**Figure 1:**
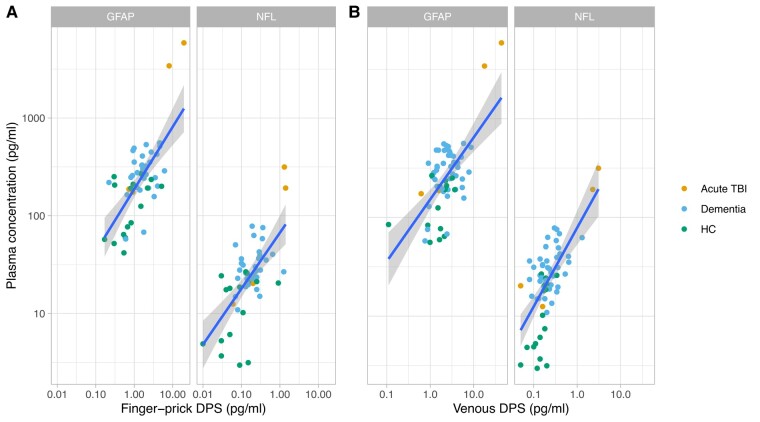
**Relationship of plasma biomarker concentrations to finger-prick and venous dried plasma spot measurements.** Correlation between gold-standard plasma sampling and finger-prick samples using dried plasma spots for glial fibrillary acidic protein (GFAP, left panel) and neurofilament light (NfL, right panel). Each participant is represented by a single point, with patient/control group is indicated by colour. Linear regression line is shown in blue with 95% confidence interval in grey. Significant positive correlations were present for GFAP (***ρ***=0.57; *P* < 0.001) and NfL (***ρ*** = 0.58; *P* < 0.001) using finger-prick. Correlations between plasma samples and venous blood on DPS showed were similar in respect of NfL but weaker for GFAP (NfL ***ρ*** = 0.57; GFAP ***ρ*** = 0.43; *P* < 0.001). TBI: traumatic brain injury. HC, healthy control; DPS, dried plasma spot; Conc., concentration.

Next, we tested the biomarker correlations between plasma and both finger-prick and venous DPS samples, by individual (Spearman’s correlation, Fig. 1, Supplementary Fig. 1). Significant positive correlations were present for GFAP (***ρ*** = 0.57; *P* < 0.001) and NfL (***ρ*** = 0.58; *P* < 0.001) using finger-prick. Correlations between plasma samples and venous blood on DPS showed were similar in respect of NfL but weaker for GFAP (NfL ***ρ*** = 0.57; GFAP ***ρ*** = 0.43; *P* < 0.001).

There was a significant decrease in levels of capillary GFAP (17.9% per month (95% CI 9.3–25.7) *P* < 0.001) and NfL (14.0% per month (95% CI 3.7–23.3) *P* < 0.05) taken from the DPS, when plotted against storage time. There was also a significant decrease for venous DPS GFAP and NfL, but not with the standard plasma samples (Supplementary Fig. 2).

**Figure 2:**
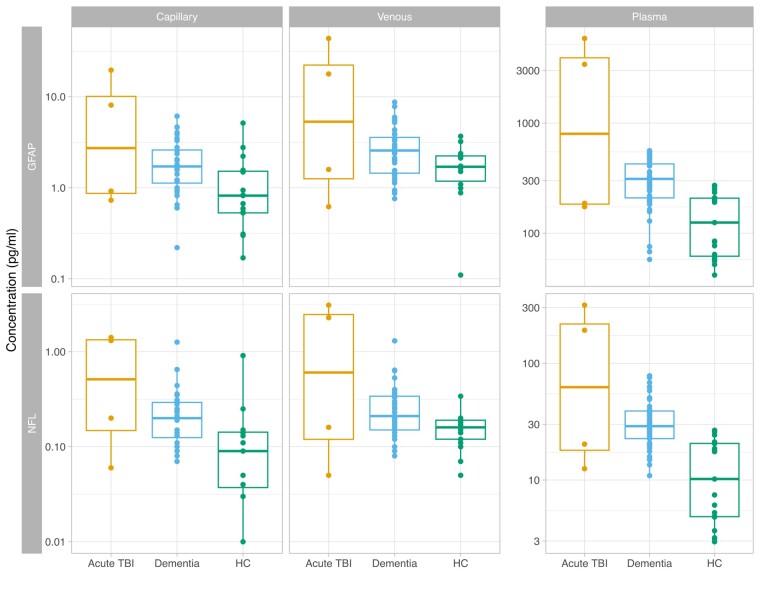
**Biomarker concentrations in different sample types by participant group.** Concentrations of GFAP (glial fibrillary acidic protein, top row) and NfL (neurofilament light, lower row), with grey columns denoting sample type. Acute TBI (traumatic brain injury) patients shown in orange, dementia in blue and healthy controls in green. Separate y-axis is used for plasma due to much higher concentrations compared to DPS (dried plasma spot) results. Linear regression was used to test differences in concentration by participant group, with concentration log-transformed and with age as a confounder. Versus healthy controls, plasma GFAP is significantly raised in both acute TBI (1280% increase, 95%CI 548-2830, *P* < 0.001) and dementia (53.1% [1.45-131], *P* = 0.042). Finger-prick DPS GFAP was raised in the TBI group only (610% increase [163–1820], *P* < 0.001); and on venous DPS significant GFAP elevation was only seen in TBI (475% increase [111–1470], *P* < 0.001). Plasma NfL was elevated in both acute TBI (1350% increase [95% CI 655–2680, *P* < 0.001]) and dementia (67.3% increase [17.0–139], *P* = 0.005) compared with healthy controls. Finger-prick NfL was elevated in acute TBI only (939% higher [95% CI 266–2850] *P* < 0.001). Venous DPS NfL was elevated after TBI (elevated by 332% [90–881] *P* < 0.001) but not in people with dementia. Boxplots show median and interquartile ranges with individual data points plotted. HC, healthy control.

Next, we tested the separation of the patient groups using different fluid samples (i.e. Plasma, finger-prick and capillary DPS). Plasma GFAP concentrations were significantly raised in both acute TBI (1280% increase, 95%CI 548–2830, *P* < 0.001) and dementia (53.1% [1.45–131], *P* = 0.042), compared with healthy controls. On finger-prick DPS testing, GFAP was significantly raised only in the TBI group (610% increase [163–1820], *P* < 0.001) and likewise on venous DPS testing, significant elevation was only seen in TBI (475% increase [111–1470], *P* < 0.001) (Fig. 2).

Plasma NfL was significantly elevated in both acute TBI (1350% increase [95% CI 655–2680, *P* < 0.001]) and dementia (67.3% increase [17.0–139], *P* = 0.005) compared with healthy controls. On finger-prick NfL testing, concentrations were significantly elevated in acute TBI (939% higher [95% CI 266–2850] *P* < 0.001) but a dementia-associated increase was not significant, versus controls. This was also the case on venous DPS NfL testing where elevation was significant in patients with TBI (increase by 332% [90–881] *P* < 0.001) but not in people with dementia.

## Discussion

In this study, we show significant moderate positive correlations of finger-prick blood sampling and gold-standard plasma quantification of GFAP and NfL in a clinical cohort, comprising patients with dementia and TBI, as well as healthy volunteers. We show clear differentiation of the acute TBI group using finger-prick GFAP and NfL, and a non-significant trend towards separation in the dementia group.

This work extends two previous investigations, both of which looked specifically at NfL using DPS. The first examined NfL concentrations in an unselected population^11^ which showed that venous DPS results were very strongly correlated with plasma, where another group showed correlations between plasma and venous DPS NfL in ALS.^12^ Showing that GFAP can be accurately quantified with finger-prick DPS, alongside NfL, has significant implications. For example, GFAP is elevated earlier in the Alzheimer’s disease process than NfL^7^ and in TBI, GFAP peaks within hours-to-days after ictus, much earlier than NfL, which reaches maximal levels around three weeks post-injury.^4^

We found concentrations were substantially elevated in plasma compared with DPS, by around 100 times. The cause of this large difference is uncertain but likely reflects technical factors. Questions remain about the effect of the DPS technique on analyte concentrations, since our results show differing correlations using venous blood applied to the DPS cards versus with finger-prick blood, compared with plasma. The volume of blood collected, amount of finger-tip squeezing, and contamination by substances of the surface of the skin, are all potential confounders.^21^ There is also a need to establish whether bespoke cut-off values for clinical abnormality should be employed when using DPS.

Finger-prick blood sampling using DPS has a range of advantages over traditional approaches. Firstly, it negates the need for a trained clinician to perform venepuncture, which is relatively time-consuming and expensive, and requires specialist equipment. It also offers the opportunity of collection of samples autonomously by patients at home and repeatedly over time. However, this was not tested in this study. Unlike the pre-processing of traditional blood samples, DPS does not require immediate transport to a laboratory for centrifuging and, whilst NfL in venous blood has been shown to be stable for 24 h at room temperature,^10^ DPS samples may be stable at room temperature for even longer when collected this way. These inherent advantages enable a broad range of uses in clinical care. There is a rapidly increasing interest in using blood biomarkers for diagnosis and longitudinal tracking of disease progression in TBI and dementia.^1^ This has growing relevance given that disease-modifying treatments are now available and best deployed in the earliest stages. Clinical trials in neurology also have a strong need of valid surrogate biomarker outcomes indicative of pathology and therapeutic response.^22^

We saw a significant decrease in levels of capillary and venous GFAP and NfL taken from the DPS, when plotted against storage time, that was not seen with the standard plasma samples (Supplementary Fig. 2). This may have contributed to the lower strength of correlations with plasma results seen in our paper compared to previous work and there is a need to further establish the best storage protocol.

There are several potential limitations. The correlation between biomarker quantification in plasma and finger-prick DPS was imperfect, being moderate in magnitude. We were unable to fully explain this, although there are several possible sources of bias and noise^12^ which may be clarified and better mitigated as the method is refined. Second, there remain questions regarding the ability of patients, particularly those with cognitive impairment and dexterity issues, to administer the tests themselves, without supervision. Therefore, collection devices that are more user-friendly need to be investigated in a variety of patient groups, or with appropriate caregiver instructions. Third, in order to rely on this technology for the means of detecting pathology, there is a need to establish normative values. This can be achieved through a more extensive data collection from healthy people across the lifespan to plot age curves, and/or to establish a robust conversion of values acquired by DPS to the equivalent as measured by standard blood tests. Fourth, although measuring duplicates would allow us to produce more reliable results and ascertain the degree of variance between replicates, these would require more sample volume than singlicates and hence greater dilution, resulting in loss of the signal. Future studies may address this problem by collecting multiple cards per participant to allow for duplicate analysis. Even the results that are below the established lower limit of quantification are above the limit of detection and perhaps new, lower cut-offs for quantification would be appropriate in the context of DPS samples given that significant correlations remain at these low concentrations. Therefore, whilst interpretation of the results in terms of absolute quantification is limited, they are still meaningful in terms of establishing a correlation with values obtained from standard plasma sampling. Fifth, finger-prick capillary blood contains some arterial as well as venous blood, and this may limit correlations with purely venous blood. We are engaged with collaborators in investigating results from arterial blood in future work. Finally, whilst we were sufficiently powered to conduct this analysis with the sample size used, there is a need to attempt to replicate the findings from this pilot study in a larger cohort.

In conclusion, we show that that GFAP and NfL can be accurately quantified using finger-prick blood sampling with DPS cards. Overall, this technique has large potential for impact across a range of neurological disorders including dementia and TBI, potentially widening access to diagnosis, disease monitoring and outcome assessment. Future work might extend these analyses to include other biomarkers, such as disease-specific molecules (e.g. phosphorylated tau in AD), other neurological diseases (e.g. inflammatory diseases such as multiple sclerosis) and systematically study the effects of different handling/storage protocols to further optimize accuracy.

## Supplementary Material

fcae151_Supplementary_Data

## Data Availability

The data are available from the corresponding author upon reasonable request.

## References

[1] Zetterberg H, Burnham SC. Blood-based molecular biomarkers for Alzheimer’s disease. Mol Brain. 2019;12(1):26. 30922367 10.1186/s13041-019-0448-1 PMC6437931

[2] Ashton NJ, Janelidze S, Al Khleifat A, et al A multicentre validation study of the diagnostic value of plasma neurofilament light. Nat Commun. 2021;12(1):3400. 34099648 10.1038/s41467-021-23620-z PMC8185001

[3] Shahim P, Politis A, van der Merwe A, et al Time course and diagnostic utility of NfL, tau, GFAP, and UCH-L1 in subacute and chronic TBI. Neurology. 2020;95(6):e623–e636. 32641529 10.1212/WNL.0000000000009985 PMC7455355

[4] Graham NSN, Zimmerman KA, Moro F, et al Axonal marker neurofilament light predicts long-term outcomes and progressive neurodegeneration after traumatic brain injury. Sci Transl Med. 2021;13(613):eabg9922. 10.1126/scitranslmed.abg9922 34586833

[5] Ashton NJ, Moseby-Knappe M, Benedet AL, et al Alzheimer disease blood biomarkers in patients with out-of-hospital cardiac arrest. JAMA Neurol. 2023;80(4):388–396. 36877496 10.1001/jamaneurol.2023.0050 PMC9989959

[6] Meier S, Willemse EAJ, Schaedelin S, et al Serum glial fibrillary acidic protein compared with neurofilament light chain as a biomarker for disease progression in multiple sclerosis. JAMA Neurol. 2023;80(3):287–297. 36745446 10.1001/jamaneurol.2022.5250 PMC10011932

[7] Benedet AL, Mila-Aloma M, Vrillon A, et al Differences between plasma and cerebrospinal fluid glial fibrillary acidic protein levels across the Alzheimer disease Continuum. JAMA Neurol. 2021;78(12):1471–1483. 34661615 10.1001/jamaneurol.2021.3671 PMC8524356

[8] Montoliu-Gaya L, Alcolea D, Ashton NJ, et al Plasma and cerebrospinal fluid glial fibrillary acidic protein levels in adults with down syndrome: A longitudinal cohort study. EBioMedicine. 2023;90:104547. 37002988 10.1016/j.ebiom.2023.104547 PMC10070083

[9] O'Connor A, Abel E, Benedet AL, et al Plasma GFAP in presymptomatic and symptomatic familial Alzheimer's disease: A longitudinal cohort study. J Neurol Neurosurg Psychiatry. 2023;94(1):90–92. 35948396 10.1136/jnnp-2022-329663 PMC9763156

[10] Verberk IMW, Misdorp EO, Koelewijn J, et al Characterization of pre-analytical sample handling effects on a panel of Alzheimer's disease-related blood-based biomarkers: Results from the standardization of Alzheimer's blood biomarkers (SABB) working group. Alzheimers Dement. 2022;18(8):1484–1497. 34845818 10.1002/alz.12510 PMC9148379

[11] Simrén J, Ashton NJ, Blennow K, Zetterberg H. Blood neurofilament light in remote settings: Alternative protocols to support sample collection in challenging pre-analytical conditions. Alzheimers Dement (Amst). 2021;13(1):e12145. 33665338 10.1002/dad2.12145 PMC7896630

[12] Lombardi V, Carassiti D, Giovannoni G, Lu C-H, Adiutori R, Malaspina A. The potential of neurofilaments analysis using dry-blood and plasma spots. Sci Rep. 2020;10(1):97. 31919375 10.1038/s41598-019-54310-y PMC6952412

[13] McKhann G, Drachman D, Folstein M, Katzman R, Price D, Stadlan EM. Clinical diagnosis of Alzheimer's disease: Report of the NINCDS-ADRDA work group under the auspices of department of health and human services task force on Alzheimer's disease. Neurology. 1984;34(7):939–944. 6610841 10.1212/wnl.34.7.939

[14] Mioshi E, Dawson K, Mitchell J, Arnold R, Hodges JR. The addenbrooke's cognitive examination revised (ACE-R): A brief cognitive test battery for dementia screening. Int J Geriatr Psychiatry. 2006;21(11):1078–1085. 16977673 10.1002/gps.1610

[15] Rosen WG, Mohs RC, Davis KL. A new rating scale for Alzheimer's disease. Am J Psychiatry. 1984;141(11):1356–1364. 6496779 10.1176/ajp.141.11.1356

[16] Caro J, Ward A, Ishak K, et al To what degree does cognitive impairment in Alzheimer's disease predict dependence of patients on caregivers? BMC Neurol. 2002;2(1):6. 12184819 10.1186/1471-2377-2-6 PMC123722

[17] Malec JF, Brown AW, Leibson CL, et al The mayo classification system for traumatic brain injury severity. J Neurotrauma. 2007;24(9):1417–1424. 17892404 10.1089/neu.2006.0245

[18] Kim JH, Woenker T, Adamec J, Regnier FE. Simple, miniaturized blood plasma extraction method. Anal Chem. 2013;85(23):11501–11508. 24156552 10.1021/ac402735y

[19] Faul F, Erdfelder E, Buchner A, Lang AG. Statistical power analyses using G*power 3.1: Tests for correlation and regression analyses. Behav Res Methods. 2009;41:1149–1160. 19897823 10.3758/BRM.41.4.1149

[20] Giebel CM, Challis D. Sensitivity of the Mini-mental state examination, Montreal cognitive assessment and the Addenbrooke's cognitive examination III to everyday activity impairments in dementia: An exploratory study. Int J Geriatr Psych. 2017;32(10):1085–1093. 10.1002/gps.4570 27593974

[21] Crimmins EM, Zhang YS, Kim JK, et al Dried blood spots: Effects of less than optimal collection, shipping time, heat, and humidity. Am J Hum Biol. 2020;32(5):e23390. 31922324 10.1002/ajhb.23390 PMC7347424

[22] Graham NS, Sharp DJ. Understanding neurodegeneration after traumatic brain injury: From mechanisms to clinical trials in dementia. J Neurol Neurosurg Psychiatry. 2019;90(11):1221–1233. 31542723 10.1136/jnnp-2017-317557 PMC6860906

