# Supplementary material for: Quantification of neurofilament light and glial fibrillary acidic protein in finger-prick blood
Source: Brain Commun. 2024 Apr 29;6(3):fcae151. doi: 10.1093/braincomms/fcae151 (PMC11189302; doi:10.1093/braincomms/fcae151)
Supplement: fcae151_Supplementary_Data [file fcae151_supplementary_data.pdf]

SUPPLEMENTARY MATERIALS

Supplementary Figure 1. **Correlations between fluid biomarkers of neurodegeneration and astroglial activation**

Spearman's correlations shown between neurofilament light (NfL) and glial fibrillary acidic protein (GFAP) tested in different fluids. Venous and finger-prick samples are using dried plasma cards (DPS). Correlation coefficients (rho) are shown in black text and indicated by colour-bar, with dark blue for perfect positive correlation, and dark red for perfect negative correlation. Insignificant correlations at a threshold of P>0.05 are shown crossed-out in grey. All significant correlations (ie non-crossed) are at P<0.001.

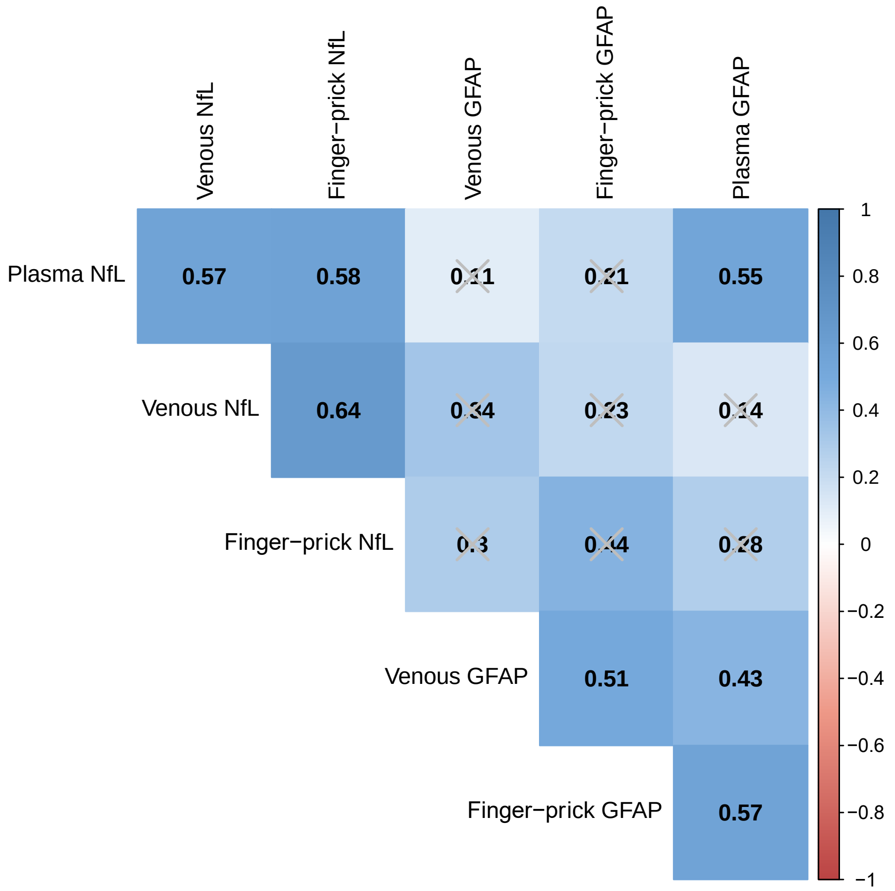

Supplementary Figure 2. **Correlations between fluid biomarkers of neurodegeneration and storage duration**

There is a significant relationship of time for each of neurofilament light (NfL) and glial fibrillary acidic protein (GFAP) in capillary dried plasma cards (DPS) and venous DPS (accounting for age), with concentrations going down with time in all. Association of biofluid concentrations and time since sampling was tested using linear regression. For capillary DPS NfL **(A)** this is a reduction in concentration of 14.0% per month (95% CI 3.7 to 23.3) P<0.05 and for capillary DPS GFAP **(B)** this is a reduction in concentration of 17.9 % per month (95 % CI

9.3 to 25.7) P<0.001. For venous DPS NfL **(C)** this is a reduction in concentration of 11.7% per month (95% CI 3.9 to 18.8) P<0.01 and for venous DPS GFAP **(D)** this is a reduction in concentration of 11.0 % per month (95 % CI 4.2 to 17.3) P<0.001. In plasma, there is no change of neurofilament light (NfL) **(E)** or glial fibrillary acidic protein (GFAP) **(F)** concentration with time since sampling (P>0.05).

A

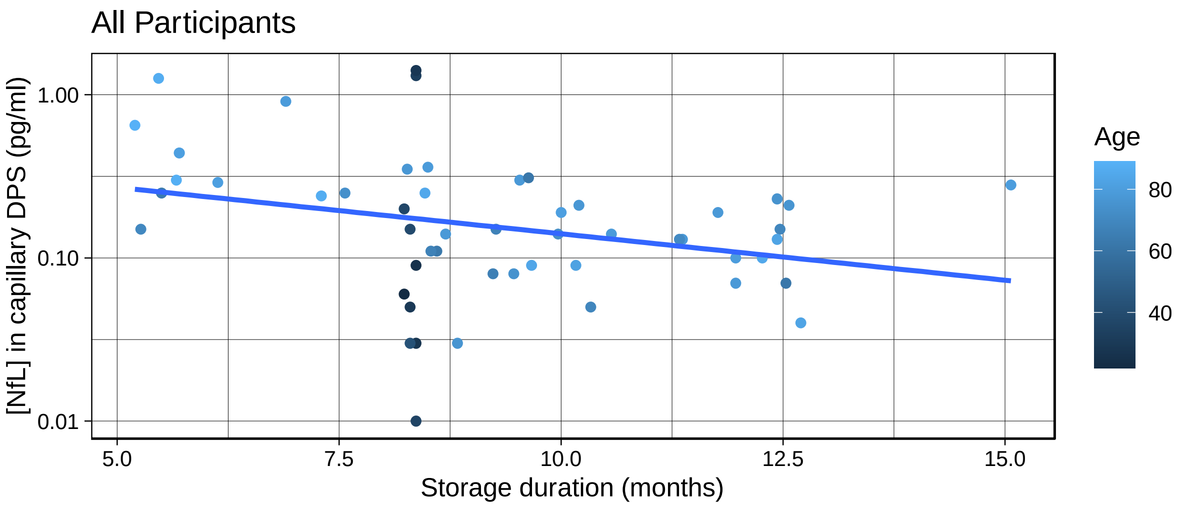

B

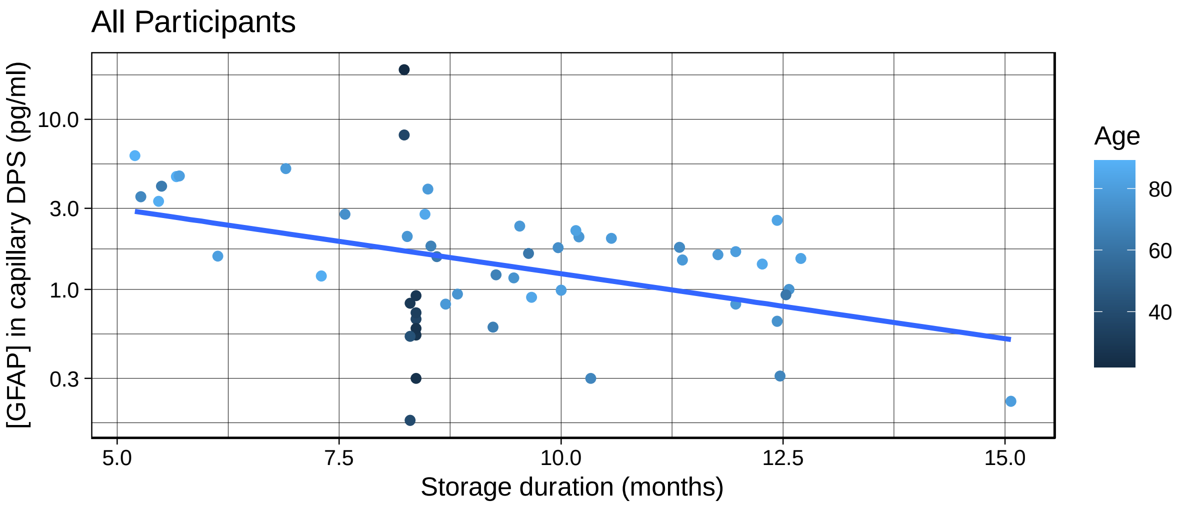

C

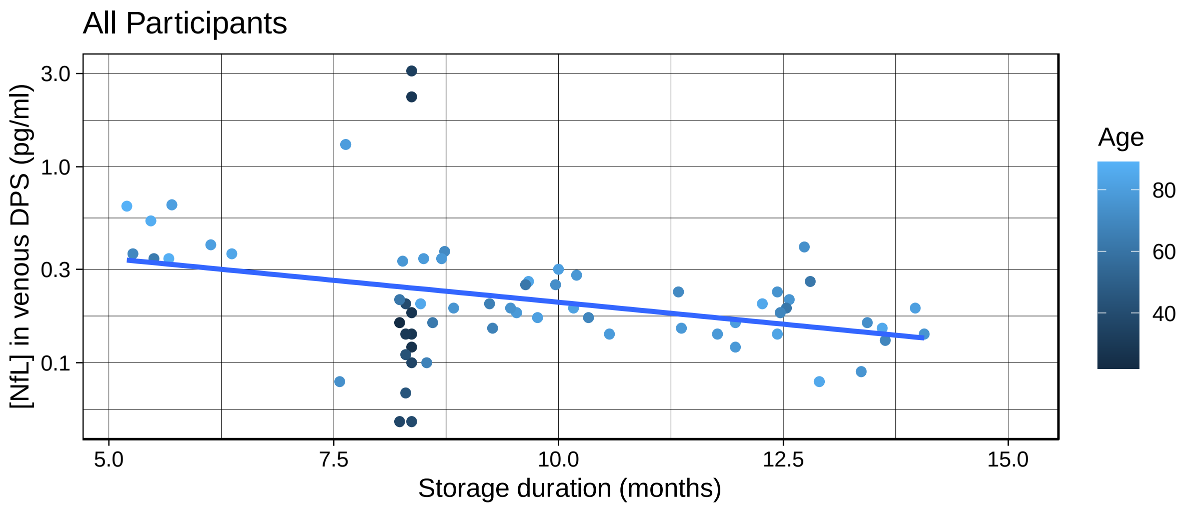

All Participants
[NfL] in venous DPS (pg/ml)
3.0
1.0
0.3
0.1
5.0
7.5
10.0
12.5
15.0
Storage duration (months)
Age
80
60
40

D

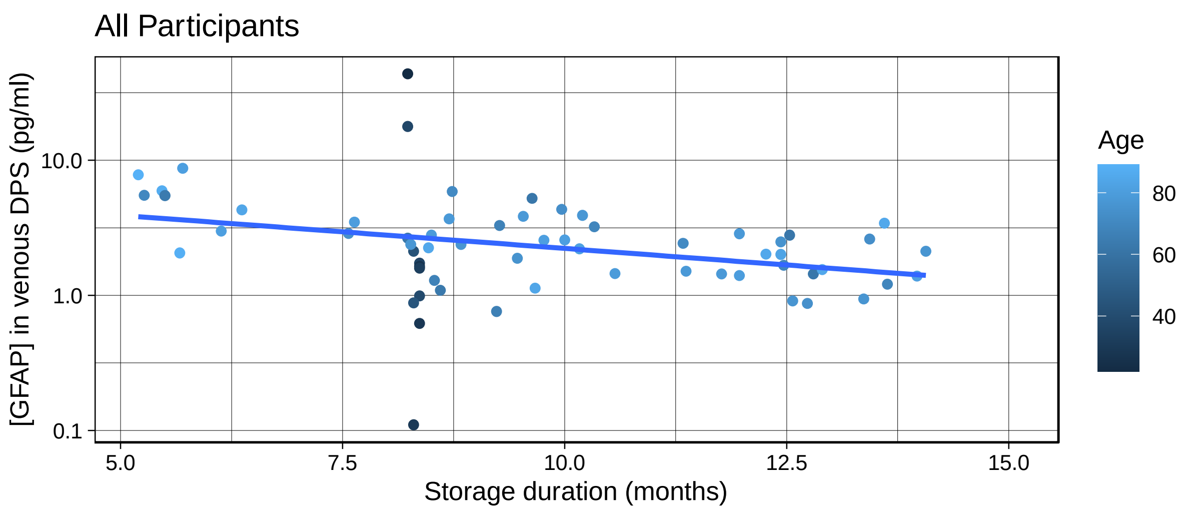

All Participants
[GFAP] in venous DPS (pg/ml)
10.0
1.0
0.1
5.0
7.5
10.0
12.5
15.0
Storage duration (months)
Age
80
60
40

E

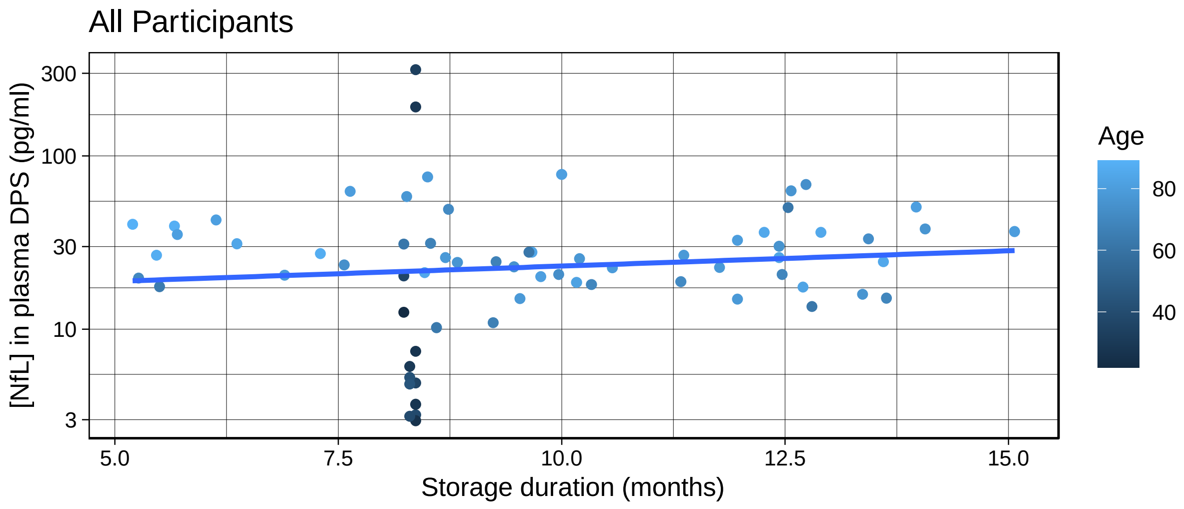

All Participants
[NfL] in plasma DPS (pg/ml)
300
100
30
10
3
5.0
7.5
10.0
12.5
15.0
Storage duration (months)
Age
80
60
40

F

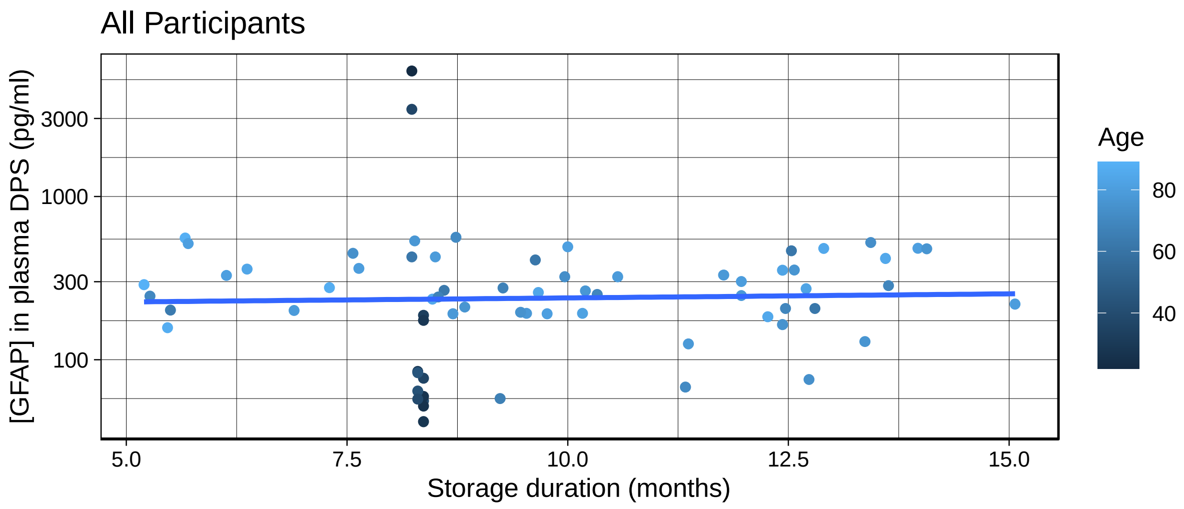

All Participants
[GFAP] in plasma DPS (pg/ml)
3000
1000
300
100
5.0
7.5
10.0
12.5
15.0
Storage duration (months)
Age
80
60
40
